# Acquired HIV drug resistance and virologic monitoring in a HIV hyper-endemic setting in KwaZulu-Natal Province, South Africa

**DOI:** 10.1186/s12981-021-00393-5

**Published:** 2021-10-16

**Authors:** Benjamin Chimukangara, Richard J. Lessells, Lavanya Singh, Indra Grigalionyte, Nonhlanhla Yende-Zuma, Rochelle Adams, Halima Dawood, Linda Dlamini, Sibonisile Buthelezi, Sheldon Chetty, Karidia Diallo, Wayne A. Duffus, Mary Mogashoa, Melissa B. Hagen, Jennifer Giandhari, Tulio de Oliveira, Pravi Moodley, Nesri Padayatchi, Kogieleum Naidoo

**Affiliations:** 1 Centre for the AIDS Programme of Research in South Africa (CAPRISA), University of KwaZulu-Natal, Durban, South Africa; 2 Department of Virology, School of Laboratory Medicine and Medical Sciences, University of KwaZulu-Natal and National Health Laboratory Service, Durban, South Africa; 3 Critical Care Medicine Department, NIH Clinical Center, Bethesda, MD USA; 4 KwaZulu-Natal Research and Innovation Platform (KRISP), College of Health Sciences, University of KwaZulu-Natal, Doris Duke Medical Research Institute, Durban, South Africa; 5 South African Medical Research Council (SAMRC), CAPRISA HIV-TB Pathogenesis and Treatment Research Unit, Durban, South Africa; 6 Infectious Diseases, Department of Medicine, Greys Hospital, Durban, South Africa; 7 National Department of Health, Pretoria, South Africa; 8 East Boom Community Health Centre, Pietermaritzburg, South Africa; 9 Division of Global HIV and Tuberculosis, Center for Global Health, Centers for Disease Control and Prevention, Pretoria, South Africa

**Keywords:** HIV-1, Acquired drug resistance, Antiretroviral treatment, Viraemia, KwaZulu-Natal

## Abstract

**Background:**

Introduction of tenofovir (TDF) plus lamivudine (3TC) and dolutegravir (DTG) in first- and second-line HIV treatment regimens in South Africa warrants characterization of acquired HIV-1 drug resistance (ADR) mutations that could impact DTG-based antiretroviral therapy (ART). In this study, we sought to determine prevalence of ADR mutations and their potential impact on susceptibility to drugs used in combination with DTG among HIV-positive adults (≥ 18 years) accessing routine care at a selected ART facility in KwaZulu-Natal, South Africa.

**Methods:**

We enrolled adult participants in a cross-sectional study between May and September 2019. Eligible participants had a most recent documented viral load (VL) ≥ 1000 copies/mL after at least 6 months on ART. We genotyped HIV-1 *reverse transcriptase* and *protease* genes by Sanger sequencing and assessed ADR. We characterized the effect of ADR mutations on the predicted susceptibility to drugs used in combination with DTG.

**Results:**

From 143 participants enrolled, we obtained sequence data for 115 (80%), and 92.2% (95% CI 85.7–96.4) had ADR. The proportion with ADR was similar for participants on first-line ART (65/70, 92.9%, 95% CI 84.1–97.6) and those on second-line ART (40/44, 90.9%, 95% CI 78.3–97.5), and was present for the single participant on third-line ART. Approximately 89% (62/70) of those on first-line ART had dual class NRTI and NNRTI resistance and only six (13.6%) of those on second-line ART had major PI mutations. Most participants (82%) with first-line viraemia maintained susceptibility to Zidovudine (AZT), and the majority of them had lost susceptibility to TDF (71%) and 3TC (84%). Approximately two in every five TDF-treated individuals had thymidine analogue mutations (TAMs).

**Conclusions:**

Susceptibility to AZT among most participants with first-line viraemia suggests that a new second-line regimen of AZT + 3TC + DTG could be effective. However, atypical occurrence of TAMs in TDF-treated individuals suggests a less effective AZT + 3TC + DTG regimen in a subpopulation of patients. As most patients with first-line viraemia had at least low-level resistance to TDF and 3TC, identifying viraemia before switch to TDF + 3TC + DTG is important to avoid DTG functional monotherapy. These findings highlight a need for close monitoring of outcomes on new standardized treatment regimens.

**Supplementary Information:**

The online version contains supplementary material available at 10.1186/s12981-021-00393-5.

## Background

The ambitious Joint United Nations Programme on HIV/AIDS (UNAIDS) 95–95–95 targets and HIV test-and-treat all approach have resulted in an increase in number of patients receiving ART [1, 2]. Despite success in reducing HIV-1 transmissions due to effective ART, some individuals develop drug resistant viruses because of poor adherence to ART or suboptimal drug concentrations, which can result for example from incorrect dosing, drug-drug interactions, and absorption problems. This is known as ADR and results in inadequate viral suppression and ongoing transmissions [3]. The World Health Organization (WHO) now recommends use of DTG a more potent integrase strand transfer inhibitor (INSTI) for HIV treatment in all ART regimens [4]. Dolutegravir has been shown to be a superior HIV drug but still has to be administered in combination with other antiretroviral drugs [5], as DTG monotherapy could result in emergence of ADR [6]. This suggests a need to maintain sensitivity to drugs used in combination with DTG to avoid DTG functional monotherapy [7].

In South Africa, adult HIV infected individuals were previously initiated on ART with a fixed dose combination of TDF plus emtricitabine (FTC) and efavirenz (EFV), whilst protease inhibitors (PIs) and INSTIs were reserved for second-line and third-line ART, respectively [8]. In line with the WHO recommendation for use of DTG, the South Africa National Department of Health in October 2019 recommended a first-line regimen fixed-dose combination of TDF plus 3TC and DTG, also known as TLD [9, 10]. The preferred second-line regimen for patients failing a first-line regimen with TDF plus 3TC and EFV (also known as TLE) is AZT plus 3TC or FTC (collectively referred to as XTC) and DTG. Patients failing a first-line regimen with AZT plus 3TC and EFV are preferably switched to a TLD second-line regimen, with protease inhibitors (PIs) being an alternative where DTG is not suitable [10].

Viral load (VL) testing remains the standard of care for monitoring patients on ART, with 2 consecutive VLs ≥ 1000 copies per millimeter (cp/mL) done at least 3 months apart (with enhanced adherence support) considered as virological failure [10]. However, patients receiving first-line DTG-based ART are considered for second-line ART only when they have at least 3 VLs ≥ 1000 cp/mL over the course of 24-months, or have other signs of immunologic or clinical failure [10]. Genotypic drug resistance testing is now also recommended (i.e. with expert advice) for patients failing first-line DTG-based ART [10]. Patients already prescribed ART without treatment failure are considered eligible for a change to a DTG-based regimen if they are considered to be ‘stable clients’, meaning those with sustained viral suppression (VLs < 50 cp/mL) on ART [10]. Despite these guidelines, there is lack of timely action on VL results, with some patients remaining on failing regimens for prolonged periods of time [11]. Given knowledge of increasing levels of pretreatment NNRTI drug resistance mutations in South Africa [12], delayed switching of patients on ART risks development and transmission of drug resistant virus, which could subsequently limit the success of DTG-based regimens in suppressing viral replication.

The first South African national drug resistance survey amongst adults failing first-line NNRTI-containing ART showed that almost all participants (96%) had drug resistant mutations after receiving ART for at least 6 months and having two consecutive VLs ≥ 1000 cp/mL [13]. The findings are broadly consistent with studies conducted specifically in KwaZulu-Natal (KZN) province in South Africa, which also showed that the majority (86–95%) of adults with virological failure on first-line ART have drug resistant mutations [14–16]. A recent nationally representative household survey of people living with HIV in South Africa also found high-levels of drug resistance to first-line ART regimens among people that were virally unsuppressed, with low major PI resistance at second-line virologic failure [17]. This again is consistent with other cross-sectional studies from South Africa which have reported lower frequencies (0–19%) of major PI mutations at second-line failure [18–20]. However, these studies did not assess the potential impact of mutations observed on susceptibility to drugs used in new DTG-based regimens.

Therefore, as part of the CAPRISA Advanced Clinical Care Programme, we conducted a study of HIV-1 ADR at the East Boom Community Health Centre, a public health facility in uMgungundlovu District Municipality, KZN, South Africa. This study aimed to determine prevalence of ADR in participants receiving ART with viraemia (i.e. having at least one VL ≥ 1000 cp/mL after receiving ART for at least 6 months) and to assess susceptibility of the virus to drugs used in combination with DTG in preferred subsequent ART regimens.

## Methods

### Study design and participant recruitment

We conducted a cross-sectional study on ADR among HIV-positive adults (≥ 18 years) accessing routine care at East Boom Community Health Centre in Pietermaritzburg, in the uMgungundlovu district (a HIV hyper-endemic setting), in central KZN, between May and September 2019. Briefly, we interrogated the routine HIV programme database (TIER.net) and weekly reports of elevated VLs from the National Health Laboratory Service to identify individuals that might be eligible to participate. At the time of searching for eligible participants in March 2019, 11,609 patients were actively receiving a documented ART regimen and 192 were eligible based on our inclusion criteria (Additional file 1: Figure S1). We tagged their clinical records at the facility and asked facility staff to refer these individuals to the research nurse at their next routine visit. On referral, we provided further information about the study and all eligible patients were offered an opportunity to participate, and if consented were enrolled in the study.

Patients were eligible if they were documented HIV-1 positive adults and currently on first, second or third-line ART for a period of at least 6 months with a latest VL ≥ 1000 copies/mL. A clinical history datasheet was completed by the research nurse for each consenting participant with information on demographic and clinical history. Following voluntary informed consent, a single venous blood sample (4 mL) was collected from each eligible participant and the samples were sent to the CAPRISA laboratory for plasma separation and then to the KwaZulu-Natal Research Innovation and Sequencing Platform (KRISP) laboratory at the University of KwaZulu-Natal, for HIV-1 drug resistance testing. Additional file 1: Figure S1 shows a summary flow diagram from participant selection to reporting of results.

### Laboratory methods

We performed HIV-1 *pol* Sanger sequencing on plasma samples using the Applied Biosystems HIV-1 Genotyping Kit according to manufacturer’s instructions [21]. In summary, we extracted viral RNA from 200 µL of pelleted plasma using a Chemagic360 platform and amplified the *protease* and *reverse transcriptase* genes according to the Applied Biosystems HIV-1 Genotyping Kit. We also amplified the integrase gene in any patients with prior INSTI exposure. We performed capillary electrophoresis on successfully amplified samples using a 3730xl DNA Analyzer (Applied Biosystems, Foster City, United States) and used the Stanford HIV drug resistance database (version 8.8) for genotypic resistance interpretation [22]. Drug resistance was defined as any NRTI, NNRTI, major PI, or INSTI resistance mutation. We defined TAMs as having any of the following classical mutations; M41L, D67N, K70R, L210W, T215FY, and K219EQ. We returned genotypic resistance test reports to the treating clinicians to support patient management.

### Data analysis

The proportions of overall ADR among participants with viraemia were estimated and the prevalence and patterns of key HIV drug resistance mutations by drug class (i.e. PI, NRTI and NNRTI) were assessed. The effects of the observed ADR mutations on predicted susceptibility to drugs used in standardized subsequent DTG-based regimens was characterized. Lastly, logistic regression was used to explore clinical variables (CD4 cell count, VL and duration on ART) and demographic variables (age and gender) associated with ADR. All statistical analyses were done using Stata v14 (StataCorp, Houston, Texas, United States). Multivariable logistic regression was performed by including all the variables in a single model.

## Results

**Fig. 1:**
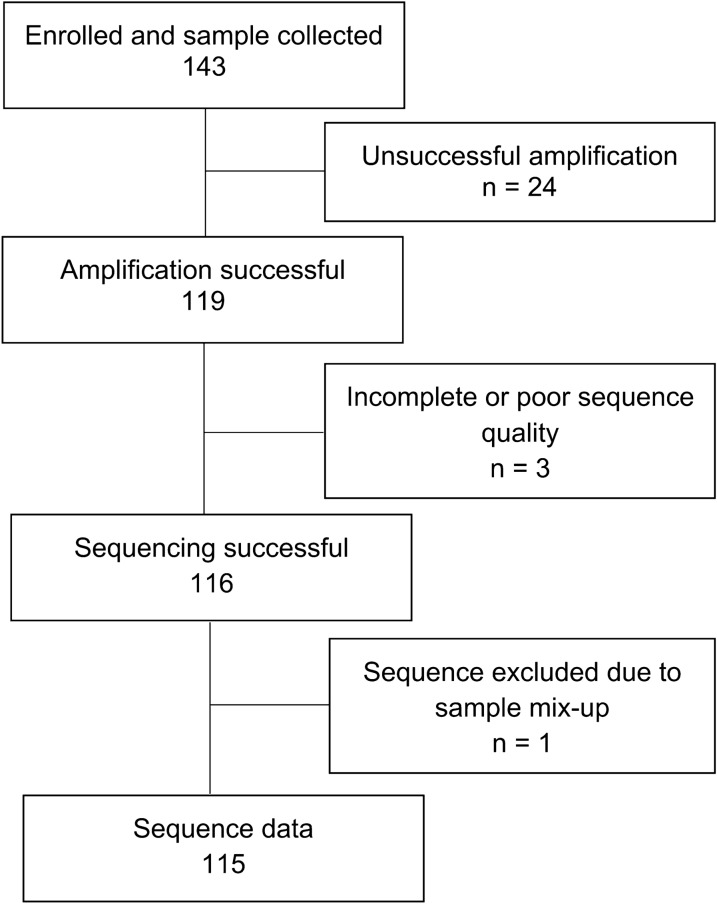
Participant and sample flow from enrolment to analysis in the acquired HIV drug resistance study in KwaZulu-Natal (KZN) province, South Africa

Of 192 patients eligible for the study, 49 could not be traced, contacted, or missed their clinic visit. Of those that could be contacted, none refused to participate or withdrew from the study. Therefore, we enrolled 143 participants between 6 May and 25 September 2019 and obtained 115 HIV-1 *pol* sequences for analysis (Fig. 1). The 28 with no sequence data had significantly lower VLs (median: 3.2 vs. 4.3 log_10_ copies/mL; p < 0.001) and higher CD4 counts (median: 529 vs. 270 cells/mm^3^, p = 0.002) at time of enrolment, compared to the 115 with sequence data (Additional file 1: Table S1). Figure 1 shows a flow chart from enrolment to samples included in final analyses.

Of the 70 participants on first-line ART, all were receiving EFV-based fixed-dose combinations, whilst most (30/44, 68%) of those on second-line ART were receiving AZT + 3TC + LPVr. The median duration on the current regimen for those on first-line ART was 24 months [interquartile range (IQR) 11–57] and the median duration on the current regimen for those on second-line ART was 21 months (IQR 7–48). Only one participant was currently on third-line ART and none of the participants were receiving a DTG-based regimen at time of enrolment. Table 1 shows the characteristics of participants enrolled in the study.

**Table 1:** Demographic and clinical characteristics of participants enrolled in the acquired HIV drug resistance study

		All participant (n = 143)	Resistance data (n = 115)	No resistance data (n = 28)
Sex, female	n (%)	76 (53.1)	62 (53.9)	14 (50.0)
Age	Median (IQR)	39 (31–46)	38 (30–46)	44 (36–50)
Current or previous TB	n (%)	64 (44.8)	56 (48.7)	8 (28.6)
Latest HIV RNA, log_10_ copies/mL	Median (IQR)	4.2 (3.4–4.8)	4.3 (3.6–4.9)	3.2 (3.1–3.6)
Latest CD4 + cell count	Median (IQR)	301 (138–429)	270 (136–394)	529 (199–728)
**ART regimen**				
First-line	n (%)	91 (63.6)	70 (60.9)	21 (75.0)
TDF + FTC +EFV	n (%)	88 (61.5)	68 (59.1)	20 (71.4)
ABC + 3TC + EFV	n (%)	3 (2.1)	2 (1.7)	1 (3.6)
Second-line	n (%)	51 (35.7)	44 (38.3)	7 (14.3)
AZT + 3TC + LPV/r	n (%)	34 (23.8)	30 (26.1)	4 (14.3)
TDF + FTC + LPV/r	n (%)	11 (7.7)	8 (7.0)	3 (10.7)
ABC + 3TC + LPV/r	n (%)	5 (3.5)	5 (4.3)	0
AZT + 3TC + ATV/r	n (%)	1 (0.7)	1 (0.9)	0
Third-line	n (%)	1 (0.7)	1 (0.9)	0
TDF + FTC + DTG + DRV/r + ETR	n (%)	1 (0.7)	1 (0.9)	0

*3TC* lamivudine, *ABC* abacavir, *ATV/r* ritonavir-boosted atazanavir, *AZT* zidovudine, *DRV/r* ritonavir-boosted darunavir, *EFV* efavirenz, *ETR* etravirine, *FTC* emtricitabine, *IQR* interquartile range, *LPV/r* ritonavir-boosted lopinavir, *TB* tuberculosis, *TDF* tenofovir

### Prevalence, patterns, and predictors of acquired drug resistance

Overall, 106/115 had at least one drug resistance mutation [92.2%, 95% confidence interval (CI) 85.7–96.4]. The proportion with resistance was similar for participants on first-line ART (65/70, 92.9%, 95% CI 84.1–97.6) and those on second-line ART (40/44, 90.9%, 95% CI 78.3–97.5). Of those on first-line ART, 62/70 (88.6%) had dual class NRTI & NNRTI resistance. The patterns of drug class-specific resistance are shown in Additional file 1: Table S2. Of those on first-line ART, 59/70 (84.3%) had the M184VI mutation associated with 3TC and FTC resistance; 31/70 (44.3%) had the K65R mutation associated with TDF resistance; and 28/70 (40.0%) had at least one thymidine analogue mutation (TAM). Of the 44 participants on second-line ART, although most had NNRTI (91%) and NRTI (82%) resistance, only six (13.6%) had at least one major PI drug resistance mutation (Table 2 and Additional file 1: Table S3). The one participant on third-line ART had triple class resistance, with NNRTI mutations (K103N, Y181C and P225H), classical TAMs (M41ML, L210W, T215F, K219R), a PI major mutation (M46I), and no INSTI resistance mutations. In the multivariable model, older participants (odds ratio (OR) 0.91, 95% CI 0.83–0.99) and those with higher CD4 counts (OR 0.82, 95% CI 0.69–0.96) had significantly reduced odds of the detection of drug resistance at viral sequencing, p < 0.05 (Additional file 1: Table S4). All sequences were HIV-1 subtype C.

**Table 2:** Drug class-specific resistance in participants on first and second-line ART regimens

ART regimen	n	NRTI resistance	NNRTI resistance	Major PI resistance
First-line	70	62 (88.6%)	65 (92.9%)	0
Second-line	44	36 (81.6%)	40 (90.9%)	6 (13.6%)

*ART* antiretroviral therapy, *NRTI* nucleoside reverse transcriptase inhibitor, *NNRTI* non-nucleoside reverse transcriptase inhibitor, *PI* protease inhibitor

### Potential impact of drug resistance mutation patterns on DTG-based ART

**Fig. 2:**
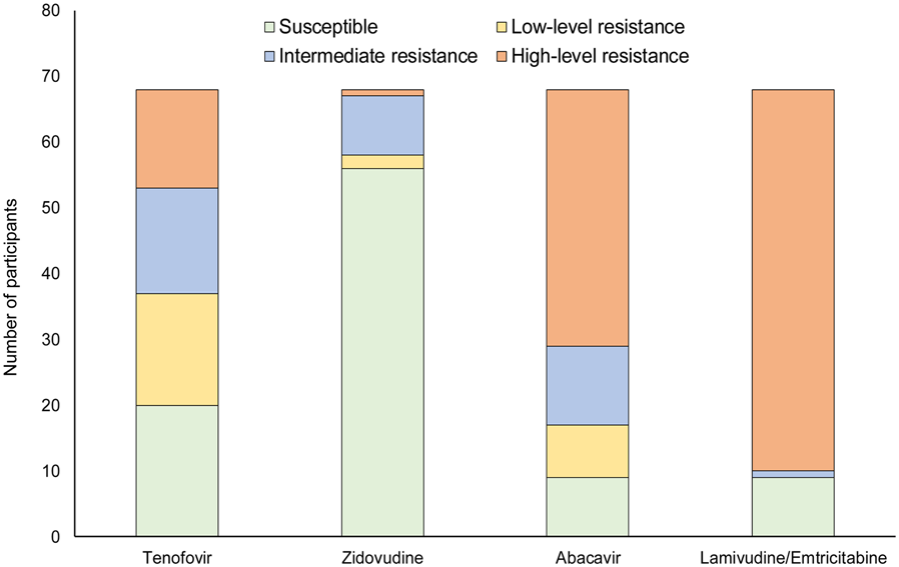
Predicted susceptibility of virus isolates from participants with viraemia receiving a tenofovir, emtricitabine and efavirenz combination regimen

Several participants with viraemia on first-line ART had atypical NRTI resistance patterns, i.e. 28/68 (41.2%) participants with viraemia on TDF + FTC + EFV had at least one TAM. In most cases (21/28) the TAMs were present without the K65R mutation; and of these cases (17/28), there was only a single TAM detected. The two most frequently observed TAMs were D67N (n = 18) and K219E/Q (n = 16). Despite this observation, in most participants with viraemia on first-line TDF + FTC + EFV (56/68, 82.4%), the virus was predicted to be susceptible to zidovudine (AZT). In contrast, the virus was predicted to be susceptible to tenofovir (TDF) in only 20 cases (29.4%) and susceptible to abacavir (ABC) in only nine cases (13.2%) (Fig. 2).

## Discussion

This study confirms findings from previous ADR surveys in South Africa showing high prevalence (86–96%) of first-line ADR [13, 15], relatively high proportions of TDF resistance (70–86%) mainly due to the K65R mutation [23, 24], and relatively low proportions of PI resistance (16–19%) at second-line failure [19, 20]. These findings support the use of AZT + 3TC + DTG as a new standardized second-line regimen for patients with first-line virologic failure. The majority (61/68) of adults with viraemia on first-line TDF + FTC + EFV had dual class (NNRTI and NRTI) resistance. In most cases, the pattern of resistance was such that the virus was predicted to be susceptible to AZT as in most cases the participants had the M184VI mutation (58/61), or the K65R mutation (30/61), or both mutations (29/61) which increase viral susceptibility to AZT. We would thus expect the new standard second-line regimen of AZT + 3TC + DTG to be effective. However, given the occurrence of atypical resistance profiles, with TAMs detected in patients on standard first-line TDF-containing regimens, there is a small group of patients for whom the standard second-line regimen of AZT + 3TC + DTG could be less effective, due to pre-existing TAMs. This suggests a need for close monitoring of VL results following medication switch to the new standardized second-line regimens, as well as investigating viral dynamics leading to atypical TAMs.

Over 40% of participants failing first-line TDF regimens already had the K65R mutation that alone causes high-level resistance to TDF and intermediate resistance to XTC [22]. If VL testing is not done or viraemia is missed, and therefore XTC ± TDF resistance is missed, this could give rise to DTG functional monotherapy and potential for emergence of DTG resistance [7]. While findings from a recent study investigating effects of recycling TDF in second-line TLD regimen and a larger randomized non-inferiority trial (i.e. NADIA trial) showed successful viral outcomes on DTG-based treatment (including in patients with prior extensive NRTI resistance), the long-term impact of pre-existing NRTI resistance on outcomes with standardised DTG-based regimens remains unclear [25, 26]. Despite high occurrence of XTC drug resistance (as shown in Fig. 2) mainly due to the M184VI mutation, use of XTC in subsequent regimens remains warranted due to the effect of the M184VI mutation on reduced HIV-1 replication capacity and increased susceptibility to AZT and TDF [27–29], the key NRTI drugs in ART regimens.

In keeping with other studies from South Africa, a minority of adults with viraemia on second-line PI-based regimens had major PI resistance mutations [19, 20]. In this study, only about one in seven patients on PI-based ART had major PI resistance mutations. This is likely to be particularly low because the median duration on second-line treatment was relatively short (less than two years), and we enrolled patients with viraemia regardless of whether they had confirmed virological failure after receiving enhanced adherence counselling. This does however highlight the ongoing challenge of viraemia without PI resistance in patients on second-line LPVr-containing regimens and the need for strategies to improve adherence and virologic suppression in this group [30]. The low prevalence of major PI resistance mutations also suggests the continued utility of PI drugs in third-line and/or salvage antiretroviral therapies.

Given that most of the participants had the NNRTI mutation K103NS (Additional file 1: Table S3) which alone does not reduce susceptibility to etravirine (ETR) a second-generation NNRTI, there remains potential utility of ETR in subsequent regimens. The use of second-generation NNRTIs in subsequent regimens, however, should be supported by genotypic resistance testing as over 90% of participants in this study harboured NNRTI mutations at second-line viraemia (Table 2). This suggests persistence of NNRTI mutations even after discontinuing treatment with NNRTI drugs [31, 32]. High rates of NNRTI resistance could also have a negative effect on the use of the investigational cabotegravir/rilpivirine (CAB/RPV), a long-acting INSTI and NNRTI combination drug. Similar to DTG use with drugs that still retain viral susceptibility, ensuring low-levels of resistance to RPV will be imperative in avoiding ‘CAB functional monotherapy’, which again could lead to drug resistance [33]. The one participant receiving TDF + FTC + DTG + DRV/r + ETR had triple class resistance (NNRTI, NRTI and PI resistance) without INSTI resistance, suggesting the continued utility of DTG/INSTIs in highly treatment experienced patients.

These findings should be interpreted with consideration of the following limitations. Basing viraemia on at least one VL result without consecutive VL measurements ≥ 1000 cp/mL done at least 3 months apart with enhanced adherence support is unconventional. However, over 90% (106/115) of the participants with viraemia in this study already had ADR mutations with 80% (92/115) having dual class NRTI- and NNRTI-resistance at time of genotyping, suggesting the need to consider early VL monitoring and switching of ART regimens. This is supported by a recent HIV Synthesis Model that showed reduction in mortality when a single VL ≥ 1000 cp/mL after at least 6-months of ART is considered a criteria for virologic failure on first-line efavirenz based treatment [34]. Secondly, we did not have adherence estimates for participants in this study. Poor ART adherence is a known contributor to development of ADR [35–37]. However, together with previous knowledge of high levels of pretreatment drug resistance in this setting [38], the high levels of ADR in this study suggest that on-going viraemia was driven by drug resistance rather than poor ART adherence alone. Ensuring high ART adherence remains important to successful viral suppression even on the new TLD regimen. Lastly, we did not get sequence data for approximately 20% of participants enrolled. This may have been partly due to the time gap between programmatic VL measurement and enrolment, such that some individuals may have achieved viral suppression or had lower VLs (i.e. VLs close to the lower limit of detection of 1000 copies/mL) at time of sample collection (Additional file 1: Table S1)*.*

## Conclusions

Resistance profiles among first-line viraemic participants with ADR suggests that the new standard second-line regimen of AZT + 3TC + DTG would be effective. However, the atypical occurrence of TAMs in TDF-treated individuals could mean a less effective AZT + 3TC + DTG regimen in a subpopulation of patients, and studies assessing mechanisms resulting in TAMs among patients not receiving thymidine analogues are warranted. Given that most patients with first-line viraemia had at least low-level resistance to TDF and 3TC, identifying viraemic patients (including those with low-level viraemia) before switching them to TLD is of vital importance to the success of subsequent ART. We believe these findings have wide relevance across South Africa and in most low and middle-income countries that follow standard HIV-1 treatment recommendations by the WHO [4]. Overall, these findings highlight the importance of vigilant monitoring of virologic outcomes, and timely genotypic drug resistance testing among patients with virologic failure on the new standardized treatment regimens.

## Supplementary Information

**Additional file 1: Figure S1.** Study flow diagram from participant selection to reporting of results in the acquired HIV drug resistance study in KwaZulu-Natal (KZN) province, South Africa, May–September 2019. **Table S1.** Comparison of participant characteristics among samples excluded and included in final analysis in the acquired HIV drug resistance study in KwaZulu-Natal (KZN) province, South Africa, May–September 2019. **Table S2.** Patterns of drug class resistance among participants on first- and second-line ART in the acquired HIV drug resistance study in KwaZulu-Natal (KZN) province, South Africa, May–September 2019. **Table S3.** Prevalence of specific acquired drug resistance mutations in the acquired HIV drug resistance study in KwaZulu-Natal (KZN) province, South Africa, May–September 2019. **Table S4.** Relationship between patient characteristics and acquired drug resistance using multivariable logistic regression.

## Data Availability

Nucleotide sequence accession numbers for HIV-1 sequences generated in this study are available from GenBank accession numbers: MW689343–MW689457.

## Abbreviations

3TC: Lamivudine
ABC: Abacavir
ADR: Acquired drug resistance
ART: Antiretroviral therapy
AZT: Zidovudine
CAB: Cabotegravir
CI: Confidence interval
cp/mL: Copies per millimeter
DTG: Dolutegravir
EFV: Efavirenz
ETR: Etravirine
FTC: Emtricitabine
INSTI: Integrase strand transfer inhibitor
IQR: Interquartile range
KRISP: KwaZulu-Natal Research Innovation and Sequencing Platform
KZN: KwaZulu-Natal
NNRTI: Non-nucleoside-reverse transcriptase inhibitor
NRTI: Nucleoside-reverse transcriptase inhibitor
PI: Protease inhibitor
RPV: Rilpivirine
TAM: Thymidine analogue mutation
TDF: Tenofovir
TLD: Tenofovir plus lamivudine and dolutegravir
TLE: Tenofovir plus lamivudine and efavirenz
VL: Viral load
WHO: World Health Organization
XTC: Lamivudine or emtricitabine
