# Supplementary material for: Acquired HIV drug resistance and virologic monitoring in a HIV hyper-endemic setting in KwaZulu-Natal Province, South Africa
Source: AIDS Res Ther. 2021 Oct 16;18:74. doi: 10.1186/s12981-021-00393-5 (PMC8520607; doi:10.1186/s12981-021-00393-5)
Supplement: Supplementary file 1 — Additional file 1: Figure S1. Study flow diagram from participant selection to reporting of results in the acquired HIV drug resistance study in KwaZulu-Natal (KZN) province, South Africa, May–September 2019. Table S1. Comparison of participant characteristics among samples excluded and included in final analysis in the acquired HIV drug resistance study in KwaZulu-Natal (KZN) province, South Africa, May–September 2019. Table S2. Patterns of drug class resistance among participants on first- and second-line ART in the acquired HIV drug resistance study in KwaZulu-Natal (KZN) province, South Africa, May–September 2019. Table S3. Prevalence of specific acquired drug resistance mutations in the acquired HIV drug resistance study in KwaZulu-Natal (KZN) province, South Africa, May–September 2019. Table S4. Relationship between patient characteristics and acquired drug resistance using multivariable logistic regression. [file 12981_2021_393_MOESM1_ESM.pdf]

SUPPLEMENTARY INFORMATION

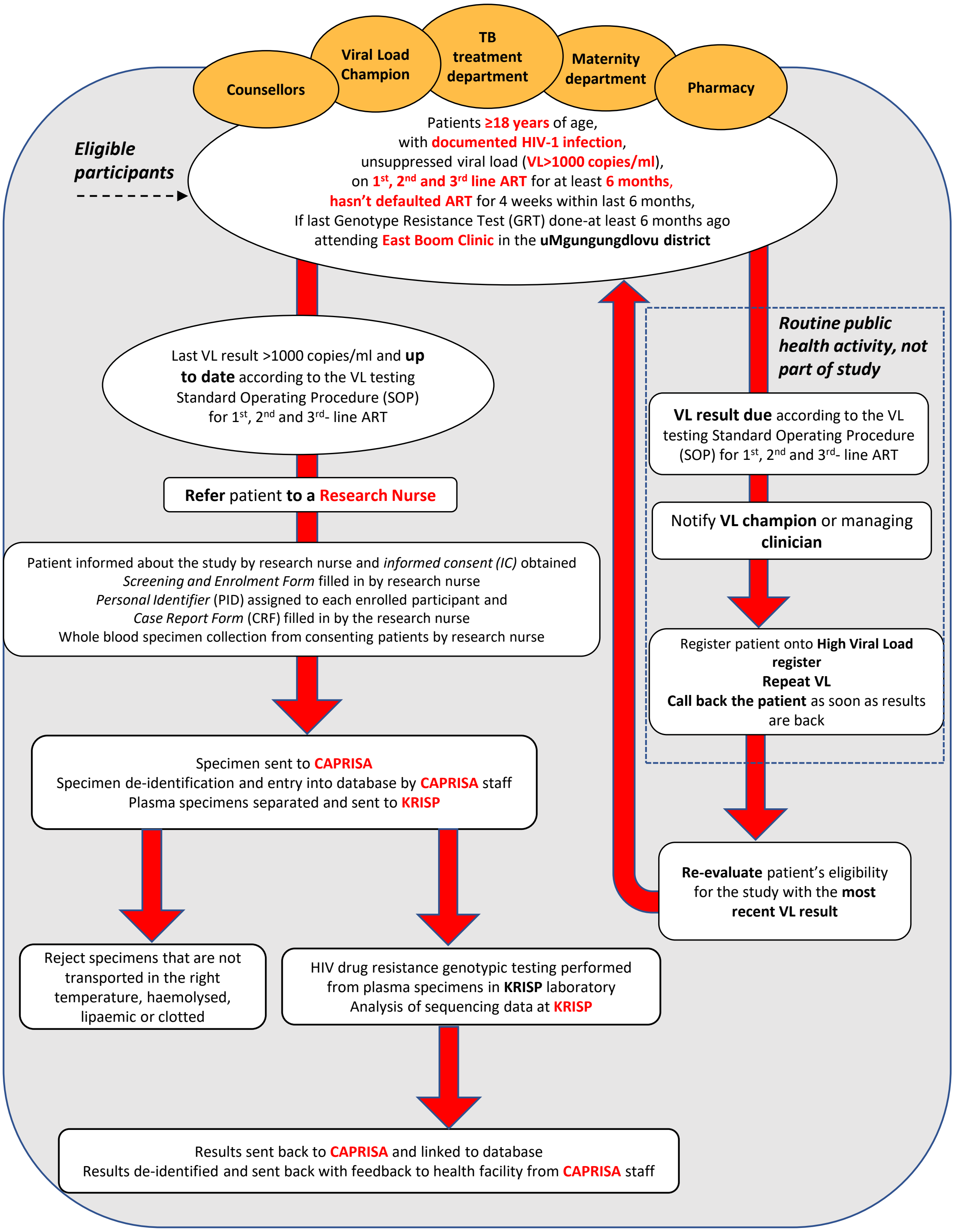

Additional file 1: Figure S1 Study flow diagram from participant selection to reporting of results in the acquired HIV drug resistance study in KwaZulu-Natal (KZN) province, South Africa, May-September 2019

Additional file 1: Table S1 Comparison of participant characteristics among samples excluded and included in final analysis in the acquired HIV drug resistance study in KwaZulu-Natal (KZN) province, South Africa, May-September 2019

Characteristic	Excluded (n=28)	Included (n=115)	p-value
Female, n (%)	14 (50)	62 (54)	0.710
Age in years, median (IQR)	44 (35-50)	38 (30-46)	0.011
Viral load (log10 copies/mL), median (IQR)	3.2 (3.0-3.6)	4.3 (3.7-4.9)	<0.001
CD4 count (cells/mm^3), median (IQR)	529 (193-737)	270 (136-394)	0.002

IQR, interquartile range; Excluded, refers to participant samples not successfully genotyped; Included, refers to participant samples that were successfully genotyped by Sanger sequencing and included in final analysis

Additional file 1: Table S2 Patterns of drug class resistance among participants on first- and second-line ART in the acquired HIV drug resistance study in KwaZulu-Natal (KZN) province, South Africa, May-September 2019

**ART regimen**	**n (%)**
First-line ART (n = 70)	
Single class resistance	*3 (4.29)*
Dual class resistance	*62 (88.57)*
Triple class resistance	*0 (0.0)*
Second-line ART (n = 44)	
Single class resistance	*4 (9.09)*
Dual class resistance	*30 (68.18)*
Triple class resistance	*6 (13.46)*

ART, antiretroviral therapy

Additional file 1: Table S3 Prevalence of specific acquired drug resistance mutations in the acquired HIV drug resistance study in KwaZulu-Natal (KZN) province, South Africa, May-September 2019

	Percentage with specific mutations		
**Mutation**	**Overall, n (%)**	**First-line ART, (n = 70)**	**Second-line ART, (n = 44)**
NNRTI mutations			
k103ns	*68 (59.13)*	*64.29*	*50.0*
v106aimt	*36 (31.30)*	*42.86*	*13.64*
g190aeq	*25 (21.47)*	*22.86*	*20.46*
v108i	*17 (14.78)*	*15.71*	*13.64*
p225hr	*17 (14.78)*	*11.43*	*18.18*
k101ehp	*16 (13.91)*	*17.14*	*9.09*
y181ci	*14 (12.17)*	*12.86*	*9.09*
v179dflt	*12 (10.44)*	*11.43*	*9.09*
e138agkq	*11 (9.57)*	*10.0*	*9.09*
y188cl	*10 (8.70)*	*7.14*	*11.36*
a98g	*9 (7.83)*	*2.86*	*15.9*
l100i	*8 (6.96)*	*8.57*	*4.55*
h221y	*8 (6.96)*	*5.71*	*9.09*
f227cl	*6 (5.22)*	*7.14*	*2.27*
m230l	*5 (4.35)*	*5.71*	*2.27*
l234i	*1 (0.87)*	*1.43*	*0*
NRTI mutations			
m184iv	*92 (80.0)*	*84.29*	*75.0*
k65enr	*39 (33.91)*	*44.29*	*18.18*
y115f	*14 (12.17)*	*15.71*	*6.82*
a62v	*13 (11.30)*	*15.71*	*4.55*
l74vi	*10 (8.70)*	*11.43*	*4.55*
k70e	*9 (7.83)*	*10.0*	*4.55*
TAMs			
k219qer	*30 (26.09)*	*28.57*	*20.46*
d67n	*25 (21.74)*	*28.57*	*11.36*
k70r	*10 (8.70)*	*8.57*	*9.09*
m41l	*9 (7.83)*	*4.29*	*11.36*
t215insyf	*6 (5.22)*	*2.86*	*6.82*
l210w	*1 (0.87)*	*0*	*0*
PI mutations			
m46i	*7 (6.09)*	*0*	*13.64*
v82a	*4 (3.48)*	*0*	*9.09*
i54v	*3 (2.61)*	*0*	*6.82*
l76v	*3 (2.61)*	*0*	*6.82*
i84v	*1 (0.87)*	*0*	*2.27*

ART, antiretroviral therapy; NNRTI, non-nucleoside reverse transcriptase inhibitor; NRTI, nucleoside reverse transcriptase inhibitor; PI, protease inhibitor; TAMs, thymidine analogue mutations

Additional file 1: Table S4 Relationship between patient characteristics and acquired drug resistance using multivariate logistic regression

Variable	OR	95% CI	p-value
Sex	1.20	0.23 – 6.18	0.829
Age (per 1-year increase)	0.91	0.83 – 0.99	0.027
CD4 count (per 50 cells/mm^3 increase)	0.82	0.69 – 0.96	0.013
Viral load (log_{10} copies/mL)	0.73	0.32 – 1.65	0.444
Duration on ART (per 1-year increase)	1.00	0.98 – 1.03	0.771

ART, antiretroviral therapy; OR, adjusted odds ratio; CI, confidence interval
